# Utilization of gamma irradiated emulsified frying oil wastes as a carbon source for sustainable and economical production of bacterial cellulose membrane

**DOI:** 10.1186/s12866-025-03931-7

**Published:** 2025-04-24

**Authors:** Doaa A. Hamed

**Affiliations:** Radiation Microbiology Department, National Center for Radiation Research and Technology (NCRRT), Egyptian Atomic Energy Authority (EAEA), Cairo, Egypt

**Keywords:** *Acinetobacterlowffii*, *Candida krusei *, SCOBY, Cooking oil, Symbiotic

## Abstract

**Background:**

Bacterial cellulose (BC) is a nanofibrils macromolecule that possesses unique properties and versatile applications in various fields. For commercial production, agro-industrial wastes were used as sustainable and cost effective alternative sources. Annually, a great amount of frying oil wastes are produced worldwide and disposed illegally resulting in huge environmental disasters. In this regard, the study aimed to investigate the effect of different concentration and increasing doses of gamma irradiation on the potential utilization of emulsified FOW as carbon source for BC production. In addition to tracking the behavior of SCOBY and BCM formation process in the presence of FOW.

**Methodology:**

The effect of different factors including; concentrations of FOW, incubation period emulsification ratios and gamma irradiation on BC production were investigated and estimated gravimetrically. In addition, the manner of the cellulose membrane formation was closely tracked and was documented by photos.

**Results:**

The data proved that the symbiotic culture (SCOBY), has the ability to utilize frying oil wastes as a sole carbon source. Addition of 1% FOW resulted in (12.1%) increasing the BCM dry weight (2.81 to 3.15 gL- 1 in SWM, while the addition of 1% of the emulsified oil (FOW/E) recording (32.6%) increase in BC dry weight compared with control (5.33 and 4.02 gL- 1, respectively). Further increase in FOW/E concentration (> 2–5%) resulted in a significant gradual decreases (39%) in BC dry weight (from 5.33 to 3.25 gL- 1). Whereas, increasing the incubation period (21- days) resulted in a significant increase in BCM dry weight from 3.79 to 5.32 gL- 1 (40.4%). The effect of gamma irradiation (0–50 kGy) of FOW/E showed an increase in BCM dry weight (2.5%) at dose 10 kGy, while recorded (34.1%) increase compared with control (without FOW). The critical moments of SCOBY while struggling for surviving to gain the oxygen and nutrients required for BC biosynthesis in the presence of FOW have been documented photographically.

**Conclusion:**

The present study positively contributes to the field of BC biosynthesis, where the FOW was added to the other agro-industrial wastes as a source of carbon for BC production, in addition to its potential application in the future in bioremediation for controlling environmental pollution.

**Supplementary Information:**

The online version contains supplementary material available at 10.1186/s12866-025-03931-7.

## Background

Green Microbiology is an interdisciplinary field that investigates microorganisms focusing on harnessing their activities and capabilities in producing or deriving value from renewable resources to provide sustainable solutions to the challenges and problems facing the environment [1]. Furthermore, microbial biotechnology plays an important role in the production and development of ecofriendly alternative biomaterials that have wide application fields. Among these biomaterials, cellulose is considered as the most abundant material along the history in which plants represent the main source of its production. However, bacterial cellulose (BC) that produced by some bacterial strains (*Acetobacter*, *Gluconobacter*, *Gluconacetobacter*, *Komagateibacter*, *Rhizobium, Sarcina*) and a symbiotic culture of bacteria and yeasts (SCOBY) has recently gained the attention due to its distinctive characteristics compared with that of plant origin [2, 3]. These bacteria commonly isolated from fermented foods such as vinegar, kombucha tea and rotting fruits are able to oxidizing sugars, alcohols and aldehydes in the presence of oxygen to acetic acid and synthesize a moist extracellular white matrix consisting of nanofibrils which called bacterial cellulose, microbial cellulose or bio-cellulose. Bacterial cellulose (BC) as the common name; is resembling hydrogels with high-water absorbency and gaseous permeability that allow the movement of nutrients required for bacterial surviving and protect them from desiccation and UV damage [4].

The global BC market is witnessing rapidly significant growth in many industries due to the increasing demand for sustainable and eco-friendly materials. According to the high cost of BC production medium which represent 30% of the total cost and considered as a barrier for its commercial scale production, the agro-industrial wastes become a novel solution to be used as carbon, nitrogen and energy sources for its production. Many studies were recommending the use of waste biomass like; wheat straw acid hydrolysate, sweet sorghum, pineapple peel, sugarcane residues, kitchen wastes and others [5–13]. In this context, using organic wastes for BC production will reduces the production cost and supports circular economy principles by converting wastes into valuable sustainable biodegradable products [14, 15]. However, using of these agro-industrial wastes require a pretreatment step through the physical, chemical, biological and thermal methods which required additional resources like chemicals and energy that will increase the net cost rather than the formation of undesirable byproducts such as phenolics that inhibit the BC synthesis [16–19].

The worldwide consumption of vegetable oils has been associated with the ascending growth of population which contribute a large amounts of frying oil wastes (FOW) annually. The direct of FOWs into water drainage; is as harmful as petroleum oil; leads to the contamination of sea and groundwater resulting in significant problems from environmental, health and economical aspects [20]. Waste oil pollution and its treatment is considered as a worldwide challenge that facing the natural resources where most of oil and grease were aggregated in high levels and enters drainage pipes causing clogging and affecting the rate of oxygen transfer in the aerobic process creating a major problem in biological system and other problems in the domestic wastewater which have serious impacts on the environment in addition to increasing the cost of treatment [21–25].

There are various pretreatment methods were used before the processing of FOWs as; *i)* removal of solid impurities by filtration, *ii)* reduction of free fatty acid (FFA) contents chemically via neutralization and esterification or physically via distillation, extraction, adsorption or membrane separation and *iii)* removal of moisture by eliminating water content to avoid hydrolysis of glycerides into FFA using steam stripping, adsorption, membrane separation and solvent extraction [26]. These methods were considered non-ecofriendly as it consumed large amounts of acids, alkalis and solvents, un-economically as it exhausting time and money or may create secondary pollution problems [18]. Whereas, biological method is one of the most promising techniques used for the removal of FFA from FOWs that demonstrate the high efficiency, safe and broad application prospects of bioremediation as a green method to solve the kitchen waste problem [27, 28]. Cooking oil which consists mainly of fatty acids or triacylglycerols can be broken down by microorganisms like bacteria, fungi and actinomycetes [29].

The previous literature mentioned several Acinetobacter species as an oil-degrading bacterium that have the capability to produce lipolytic enzymes and degrade hydrocarbons, edible and waste cooking oils [30, 31]. Most of the previous researches concluded the use of microorganisms to treat waste cooking oil and convert it into high-value resources such as bioenergy and fatty acids [32–34]. Acinetobacter strains were concluded among the unique fermentable bacteria for producing various intra- and extra- valuable products as; proteases, lipases, biocatalyst, bioemulsifiers, and biopolymers [35]. Lipases are acting on the carboxyl ester bonds present in triacylglycerols under aqueous conditions to produce fatty acids and glycerol [36]. Few reports were found which focusing on the high lipase activity of *Acinetobacter*; like *Acinetobacter haemolyticus* NS02 - 30; [23] whereas its degradation mechanism of oil has not been studied at all. Only one study was isolating *Acinetobacter junii* WCO- 9 from oil waste which illustrated a complete triglyceride degradation pathway and the specific lipase gene that has a great potential in bioremediation [28].

Vegetable oils including frying and cooking oils has been investigated to synthesis biopolymers by direct conversion or by fermentation process using different microorganisms [26]. The previous studies reported that some bacterial species have the ability to use the free fatty acids (FFA) from waste cooking oil (WCO) as a carbon source to improve its growth and in the synthesis of polyhydroxyalkanoate (PHA) which including; *Pseudomonas sp.,* and *Bacillus thermoamylovorans* [37–39].

To the best of our knowledge, this is the first study have been conducted for the production of bacterial cellulose from FOW except one study which reported that the supplementation of the production medium with 1% vegetable oil increased the BC yield over 500% [40]. In this regard, the present work aims to: *(i)*: study the potential use of frying oil wastes (FOW) as a carbon source for BC production, *(ii)*: investigate the effect of gamma irradiation pretreatment of emulsified FOW on BC production, *(iii)*: tracking the behavior of the symbiotic culture (SCOBY) during BC production and how does BCM formed along the fermentation process in the presence of emulsified and non-emulsified FOW. The study supposed that; the combination between emulsification and gamma irradiation may enhance the availability of FOW and its utilization by SCOBY for BC production.

## Materials and methods

### Chemicals

The chemicals used in HS medium for BC production and emulsification of frying oil wastes were illustrated. Yeast extract- Bacteriological Grade (Eastrin Fine Chemicals. LTD, Italy), Peptone (ADWIC, Egypt), Sodium Phosphate Dibasic Anhydrous (Fisher Scientific, China), Citric acid (Chem-Lab NV, Belgium), Glucose anhydrous (El Nasr Pharmaceutical Chemicals Co., Egypt), Tween 80 (MP Biomedicals, Inc., France), Ethanol 96% (Piochem, Egypt). Commercial sugar was used in the preparation of 6% sugared water medium (SWM) for BC production according to the previous study [41].

### Collection of FOW sample

The FOW sample was collected from a sole source of home kitchen, Cairo- Egypt to ensure the homogeneity of its components along the experiments. It is a commercial product which consists of a mixture of sunflower and soybean oil which used in frying potato for 2–3 times. A fabric textile was used to remove any cooking food residues from oil then stored at room temperature on dark for further study.

### Preparation of pre-inoculum

A symbiotic culture of bacteria and yeast (SCOBY) containing *Acinetobacter lowfi* and *Candida krusei*, respectively were isolated from commercial Kombucha beverage [41]. The inoculum culture was prepared by transferring one single colony of the SCOBY growing on HS medium [42] agar plate to a test tube containing 5 ml of HS broth medium and incubated at 30 °C for 48 h. The contents of the tube were transferred to 100 ml HS broth medium and incubated at 30 °C for 7 days under static conditions. All media under investigation were inoculated by 5% of pre-culture. HS medium consisting of (gL^−1^) glucose (20), peptone (5), yeast extract (5), dibasic sodium phosphate (2.7), citric acid (1.15) and pH was adjusted to 6.

### Quantification of BCM production gravimetrically

BCM was harvested from the media, purified by immersion in 70% ethanol for 2–3 h at room temperature in order to remove bacterial cells and any other residues, then rinsed several times with sterile distilled water. The obtained cellulose was dried at 35 ± 1 °C till constant weight and weighed on an analytical balance (Citizen Scale, CY 204, USA). BCM yield (%) was calculated according to the equation [43] with modification of the equivalent of 1 ml of FOW in grams (0.92 gm) to substitute of added to original sugar:

1 $$Yield \,of \,BCM (\%)= [BC \,dry \,wt. ({gL}^{-1})/ Original \,sugar ({gL}^{-1})] \times 100$$

The percentage of increase and decrease in BCM dry weight was calculated according to the Eq. (2 & 3):

2 $$\% \,of\, increase= [Final \,value- Initial \,value)/ initial\, value] \times 100$$

3 $$\%\, of \,decrease= [Initial \,value- Final \,value)/ initial \,value] \times 100$$

### Production of BCM at different concentrations of FOW

Different concentrations of FOW (0, 1, 2, 4 and 5%) were tested for production of BCM in both sugared and sugared free media (SWM & WM). It was inoculated, incubated statically at 30 °C for 15 days in duplicates and the dry weight of purified BCM was recorded.

### Substitution of C/N sources of HS medium by FOW

In order to confirm the ability of SCOBY to utilize FOW as C/N sources, it was added as 1% (v/v) to the HS standard BC production medium (gL^−1^) according to Table 1. The medium was inoculated with 5% pre-inoculum and incubated for 21 days at 30 °C in static conditions. BCM was harvested, immersed in 70% ethanol overnight, washed with distilled water, then dried and the dry weight was recorded.

**Table 1:** Constituents of HS medium substituted by 1% FOW

**HS (**gL^−1^**)**	**HS/FOW (I)**	**HS/FOW (II)**	**HS/FOW (III)**	**HS/FOW (IV)**	**HS/FOW (V)**
Glucose (20)	FOW (1%)	Glucose (20)	Glucose (20)	Glucose (20)	FOW (1%)
Peptone (5)	Peptone (5)	FOW (1%)	Peptone (5)	FOW (1%)	
Yeast extract (5)	Yeast extract (5)	Yeast extract (5)	FOW (1%)		
Na_2_HPO_4_ (2.7)	Na_2_HPO_4_ (2.7)	Na_2_HPO_4_ (2.7)	Na_2_HPO_4_ (2.7)	Na_2_HPO_4_ (2.7)	Na_2_HPO_4_ (2.7)
Citric acid (1.15)	Citric acid (1.15)	Citric acid (1.15)	Citric acid (1.15)	Citric acid (1.15)	Citric acid (1.15)

### Preparation of FOW emulsion (FOW/E)

FOW was emulsified using a mixture of (5%) Tween 80 and (10%) ethanol. Different oil: emulsifier and emulsifier: oil ratios (1:1, 1:2, 1:3, 1:4 & 1:1, 2:1. 3:1 and 4:1, respectively) were prepared to determine the optimum emulsification. The tubes were homogenized in vortex for 2 min and kept at room temperature for 24 h, then the height of the emulsion layer (W_e_) and the total height of the solution (W_s_) in the tube were measured and the emulsification index (EI) was calculated using the formula:

4 $${EI}_{24}= [{W}_{e} /{W}_{s}] \times 100$$

where EI_24_ is the emulsification index (EI) after 24 h at room temperature [44]. The stability of the FOW emulsion was studied along 4 weeks by calculating the EI and comparing with EI_24_.

### Production of BCM at different incubation period

The production of BCM at different incubation periods (7, 10, 15, 21 days) was investigated after addition of 1% FOW/E in sugared media (SWM). It was inoculated (5% pre-inoculum), incubated statically at 30 °C for 15 days in duplicates and the dry weight of purified BCM was recorded.

### Quantification of FOW consumption

The amount of FOW consumed was determined by separating the residual oil floating on the surface of the medium using separating funnel, then oven dried at 50 °C till constant weight. The results were expressed as a percentage of FOW consumed using the Eq. (5):

5 $$FOW consumption (\%)= ({W}_{0-} W/ {W}_{0}) \times 100$$

where W_0_ is the original weight of FOW in control vessels without inoculation and W is the weight of FOW remained on the medium at the end of the incubation period.

### Gamma irradiation of emulsified FOW and BCM production

This preliminary experiment was conducted at first, where the FOW was exposed to different gamma irradiation doses (0, 10, 20, 40, 50 kGy), then inoculated in sugar free medium (WM). It was inoculated (5% pre-inoculum), incubated statically at 30 °C for 15 days, then BCM were harvested and the data was recorded. At the end of experiments, the best result of all studied parameters was selected and 15 ml of FOW/E were exposed to different doses of gamma irradiation (0, 10, 25 and 50 kGy) in screw capped test tubes in triplicate. The Indian source (^60^Co-γ) at National Center for Radiation Research and technology (NCRRT), Cairo, Egypt (Dose rate: 0.711 kGy/h) was used. The irradiated FOW/E was added (1%) into inoculated SWM medium and incubated for 21 days. After harvesting and treatment of BCM, the dry weight was recorded as previously mentioned.

### Process of BCM formation in the presence of FOW & FOW/E

The process of BCM formation on the surface of the media in the presence of FOW and FOW/E is varied compared with control. A photo shot of the different mechanisms of BCM formation was observed, described and documented. The aim of this part was to identify the behavior of SCOBY dealing with the presence of FOW and FOW/E in both SWM and WM.

### Statistical analyses

All investigations were performed in duplicates. Data were expressed as the means ± standard deviations. Statistical differences were analyzed using one-way analysis of variance (ANOVA) by Minitab software (version 17) where the Tukey–Kramer test was used for multiple comparison of means. P-value < 0.05 considered as significant [45].

## Results

The results in Table 2, indicated the effect of different concentrations of FOW (0, 1, 2, 4 and 5%) on BCM dry weight and yield (%) in both sugared and sugared free media (SWM and WM, respectively). The addition of 1% FOW resulted in a significant increasing the BCM dry weight from 2.81 to 3.15 gL^−1^ (12.1%) with decreasing in BC yield from 4.7 to 4.6% in SWM compared with WM where the BCM dry weight increase from 0.21 to 0.27 gL^−1^ (28.6%) and increasing BC yield from 0 to 2.9%. While, increasing concentrations (2–4%) showed no significant difference of BCM dry weight in SWM compared with WM which showed a significant decrease. Whereas, a significant decrease in BCM dry weight and yield was recorded in 5% in both SWM and WM. Generally, there was an inverse relationship between the concentration of FOW and BCM dry weight, where BCM dry weight decrease as the concentration of FOW increase.

**Table 2:** BCM Dry weight of different concentrations of FOW

**Conc. of FOW (%) ml**	**BCM** **Dry weight (**gL^−1^**)**	**Yield (%)** **Sugar + FOW**	**Conc. of FOW (%) in WM**	**BCM** **Dry weight (**gL^−1^**)**	**Yield (%)** **FOW**
0	2.81 ± 0.0262 ab	4.7	0	0.21 ± 0.0019 ab	0
1	3.15 ± 0.0240 a	4.6	1	0.27 ± 0.0018 a	2.9
2	2.97 ± 0.0100 ab	3.8	2	0.18 ± 0.0004 bc	2.1
4	2.27 ± 0.0159 ab	2.3	4	0.11 ± 0.0007 c	0.298
5	1.89 ± 0.0041 b	1.8	5	0.09 ± 0.0003 c	0.195

Means with the same letters are not significantly different according to Tukey's test (P < 0.05)

SWM: Sugared water medium (6%) + FOW (ml %)

WM: Sugar free medium (water only) + FOW (ml %)

1 ml of FOW = 0.92 gm

As shown in Table 3 the obtained data proved that the SCOBY are able to utilize FOW by different levels when substitute C/N source in HS medium, even it used as a sole source of C and N in HS (V). The data revealed that the dry weight of BCM in HS (I) and (IV) media (0.24 and 1.68 gL^−1^, respectively) was less compared with HS (II) and (III) media which resulted in increasing of BCM dry weight (2.26 and 3.70 gL^−1^, respectively). However, the statistical analysis proved that HS medium (III) is not significantly differ from the control medium (HS) based on BCM dry weight (3.70 and 3.99 gL^−1^, respectively). Ultimately, the BC yield (%) in HS media containing 1% FOW substituting C/N source was lower than the original HS medium due to the difference in carbon source type and ratio.

**Table 3:** BCM Dry weight of 1% FOW substitution on HS medium

Media	HS	HS (I)	HS (II)	HS (III)	HS (IV)	HS (V)
**Sugar + FOW (**gL^−1^**)**	20 + 0%	0 + 1%	20 + 1%	20 + 1%	20 + 1%	0 + 1%
**BCM DW** **(**gL^−1^**)**	3.99 ± 0.0497 a	0.24 ± 0.0011 C	2.26 ± 0.0261 ab	3.70 ± 0.0145 a	1.68 ± 0.0324 bc	0.01 ± 0.0002 c
**Yield (%)** **Sugar + FOW**	19.95	2.6	7.7	12.7	5.8	0.12

Means with the same letters ate not significantly different according to Tukey's test (*P* < 0.05)

Composition of each medium: See Table 8 1 ml FOW = 0.96 gm

**Fig. 1:**
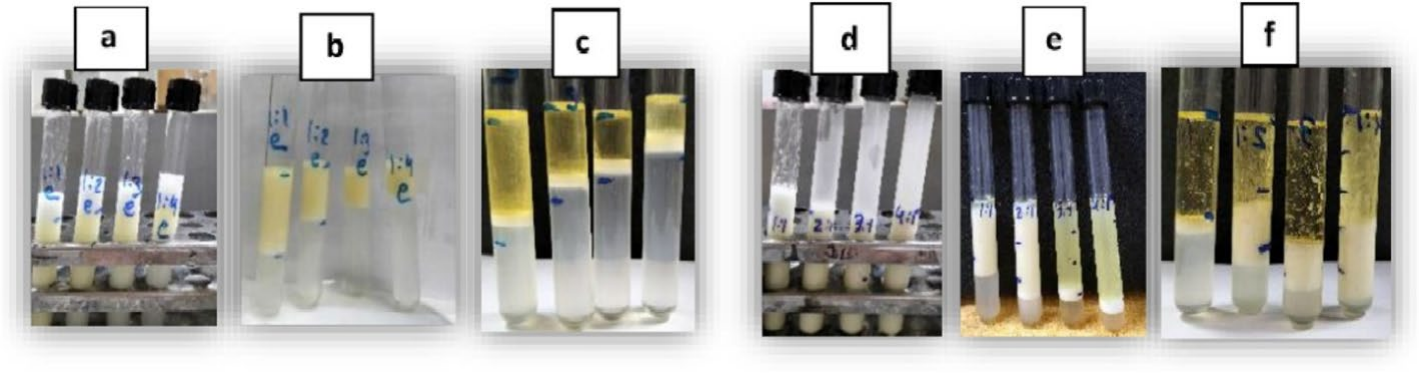
Emulsification index of different FOW: E ratios. (1:1, 1:2, 1:3 & 1:4). a: zero time, b: 24 h, c: 4 weeks and (1:1, 2:1, 3:1 & 4:1) d: zero time, e: 24 h, f: 4 weeks

In order to reach the optimum emulsification, different FOW: Emulsifier ratios (1:1, 1:2, 1:3, 1:4 & 1:1, 2:1, 3:1 and 4:1) were examined after 24 h (EI_24_) and 4 weeks (EI_f_) to determine its stability during this period. The emulsification index (EI_24_) in Table 4 showed that the ratio of 2:1 (FOW: E) was the highest (70.7%) followed by the ratio 1:1, where EI24 was 60% while the ratio of 4:1 represent the minimum value (8.0%). On the other side, the EI of all the samples recorded less values after 4 weeks, where the ratio (4:1) recorded the highest value (EI_f_ = 37.3) compared with the ratio (1:1) which recorded the lowest value (EI_f_ = 5.33). As the ratio 2:1 (FOW: E) recorded the highest high of emulsion after 24 h, subsequently it was selected for conducting the further studies after preparation with 24 h (Fig. 1).

**Table 4:** Emulsification index of different FOW: Emulsifier ratio

FOW: E	26-May	EI_24_	4 weeks	EI_f_	FOW: E	24 h	EI_24_	4 weeks	EI_f_
1:01	4.5/7.5	60	0.4/7.5	5.33	1:01	4.5/7.5	60	0.4/7.5	5.33
1:02	3.2/7.5	42.6	0.5/7.5	6.66	2:01	5.3/7.5	70.7	2.5/7.5	33.3
1:03	2.6/7.5	34.7	0.44/7.5	5.86	3:01	0.7/7.5	9.3	1.8/7.5	24
1:04	2/7.5	26.6	0.8/7.5	10.6	4:01	0.6/7.5	8	2.8/7.5	37.3

EI_24_ Emulsification index after 24 h

EI Final emulsification index (after 4 weeks)

The production of BCM at different incubation periods (7, 10, 15, 21 days) was investigated after addition of 1% FOW/E. The data (Table 5) proved that the dry weight of BCM and its yield was increased by increasing time of incubation reaching its maximum (5.32 gL^−1^ and 7.7%, respectively) after 21 days, then tends to stabilize (5.30 gL^−1^ and 7.7%, respectively). The data concluded that, the addition of 1% emulsified FOW (FOW/E) in (2:1 ratio) increase the BCM dry weight by (20.3%) compared with the non-emulsified form (3.79 and 3.15 gL^−1^, respectively). Whereas, increasing the incubation period resulted in a significant increase in BCM dry weight (from 3.79 to 5.32 gL^−1^) which representing (40.4%).

**Table 5:** Dry weight of BCM in SWM with 1% FOW/E at different incubation periods

**Incubation period (days)**	**BCM dry weight (**gL^−1^**)**	**Yield (%)**
7	1.66 ± 0.0099 d	2.4
10	2.24 ± 0.0017 c	3.2
15	3.79 ± 0.0103 b	4.6
21	5.32 ± 0.0015 a	7.7
30	5.30 ± 0.0008 a	7.7

Means with the same letters ate not significantly different according to Tukey's test (*P* < 0.05)

The addition of different concentrations of FOW/E was illustrated in Table 6, it was confirmed that the addition of 1% of FOW/E is the optimum concentration for BCM dry weight, as the increase in BCM dry weight was recorded (32.6%) compared with control (5.33 and 4.02 gL^−1^, respectively) and increasing the yield from 6.7 to 7.7%. Meanwhile, it was assumed that the graduated increase in FOW/E concentration in the presence of SWM may lead to the increase of BCM production, but it was found that a significant gradual decrease reaching (39%) was occurred in both dry weight and yield from 5.33 to 3.25 gL^−1^ and from 7.7 to 4.7, respectively).

**Table 6:** BCM dry weight of different conc. of FOW/E in SW medium

**FOW/E (%)**	**BCM dry weight (**gL^−1^**)**	**Yield (%)**
E	0.299 ± 0.0028991 d	0
0	4.02 ± 0.034648 bc	6.7
1	5.33 ± 0.0700743 a	7.7
2	4.97 ± 0.087186 a	7.2
4	4.69 ± 0.046103 ab	6.8
5	3.25 ± 0.043487 c	4.7

Means with the same letters ate not significantly different according to Tukey's test ((*P* < 0.05)

E: means addition of 1% emulsifying solvent (5% Tween 80 + 10% ethanol in water) as a control

The results in Table 7 indicated the amount of FOW and FOW/E consumed in SWM and WM for BCM production in addition to the amount which wasted during separation process. The optimum consumption (86.5%) was recorded in SWM containing FOW in its emulsified form, while the minimum consumption (39.2%) was noticed in WM containing FOW only as a sole nutrition source. Ultimately, the percentage of oil wasted during separation was ranged between 1.8- 2.9% of the total amount of FOW.

**Table 7:** Consumption of FOW & FOW/E (%) in SW & W media

**SWM**	W_0_	W	**FOW consumption (%)**	**WM**	W_0_	W	**FOW** **Consumption (%)**
FOW*	2.73	2.66	2.6	FOW*	2.73	2.65	2.9
FOW/E*	2.76	2.69	2.5	FOW/E*	2.76	2.71	1.8
FOW	2.65	0.85	67.9	FOW	2.68	1.63	39.2
FOW/E	2.67	0.36	86.5	FOW/E	2.71	1.57	42.1

^*^: control without inoculation

W_0_: original dry weight of FOW in control vessels (*: without inoculation)

W: dry weight of remaining FOW after incubation period

The effect of gamma irradiation of the emulsified FOW on BCM production at different doses was investigated (Table 8). There was a significant difference in BCM dry weight between irradiated and non-irradiated FOW/E compared with the control, although the yield was not highly affected and fluctuated at the same range. The increase in BCM dry weight was just (2.5%) at dose 10 kGy compared with 0 kGy, while recorded (34.1%) increase compared with control (SWM without FOW/E). However, it was concluded that the irradiation doses may affect the FOW/E which need further study.

**Table 8:** Effect of gamma irradiated FOW/E on BCM production

**Irradiation doses (kGy)**	**BCM Dry wt. (**gL^−1^**)**	**Yield (%)**
SWM	4.05 ± 0.007 d	6.75
0	5.30 ± 0.015 b	7.66
10	5.43 ± 0.009 a	7.85
25	5.17 ± 0.006 c	7.47
50	5.21 ± 0.007 bc	7.53

*SWM* sugared water medium + SCOBY (control)

**Fig. 2:**
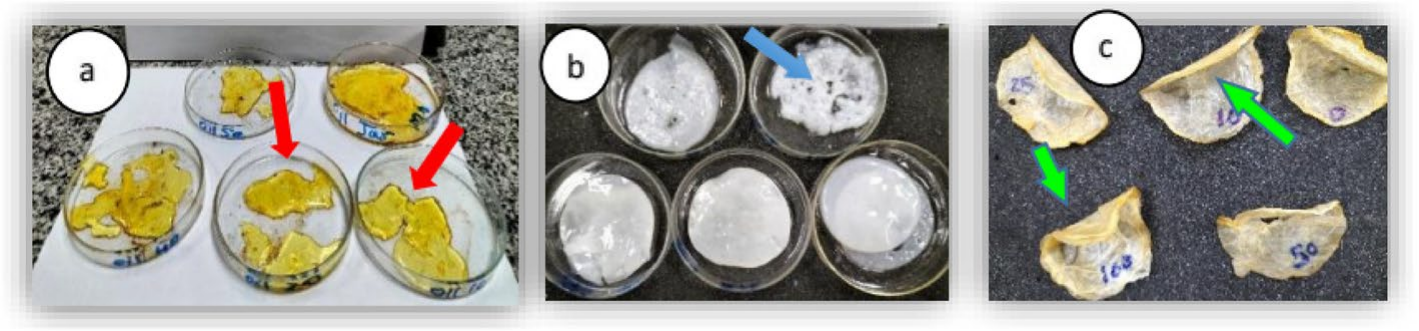
Different BCM produced: **a** oil aggregates like elastic rubber membrane (red arrows) in addition of 3.3% irradiated FOW. **b** Wet BCM at different FOW concentrations in SWM showing holes and cavities (blue arrow). **c** Dry BCM at different doses of irradiated FOW/E, the green arrows show its elasticity and how it can be folded

The variations among BCM formed at different states were illustrated in Fig. 2, where the wet and dry BCM was observed (Fig. 2b & c, respectively). In addition to the effect of high FOW: E ratio (10:1) on BCM (data not presented) where holes and cavities were appeared on the surface (blue arrow in Fig. 2b). Whereas, BCM appeared as oil aggregates like a sticky elastic film (Fig. 2a) by using irradiated FOW (0,10, 20, 40, 50 kGy) in WM at 3.3% as a preliminary experiment.

**Fig. 3:**
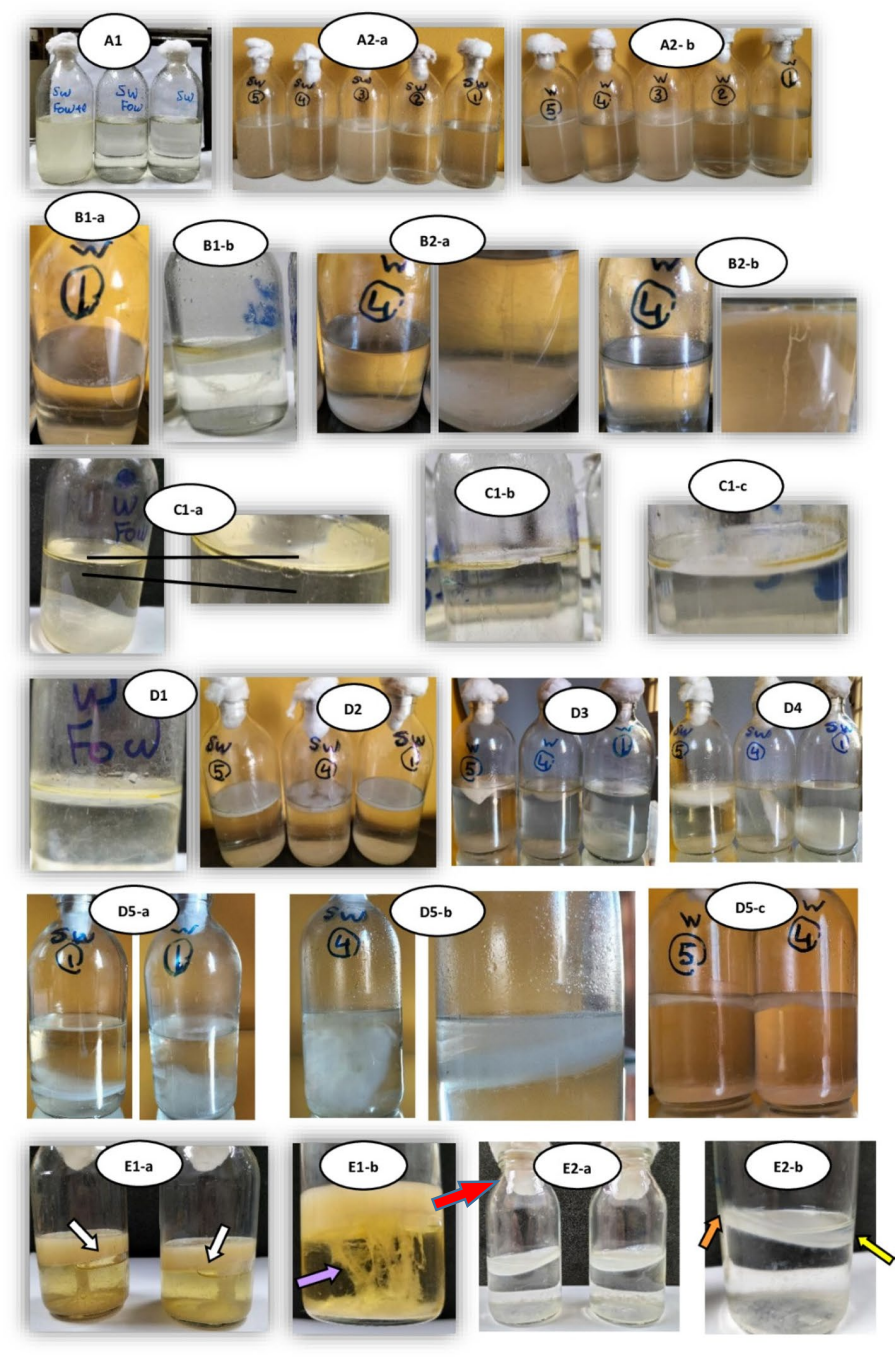
A photo shot documentation of the of BCM formation process in the presence of FOW and FOW/E in both SWM and WM during 21 days and 3 months of incubation. SWM: sugared water medium (6 % sugar)- WM: sugar free medium (dist. H_2_O)- 1: Control (SCOBY only)- 2: addition of 1% FOW- 3: addition of 1% FOW/E- 4: addition of 1% FOW+ 5% SCOBY- 5: addition of 1% FOW/E+ 5% SCOBY

The process of BCM formation on the surface of the SWM and WM in the presence of FOW and FOW/E were observed and captured along the incubation period which exhibited different characters as shown in Fig. 3 and the comparison among them was illustrated in table (9) which attached as additional file (1). The data displayed the appearance of the media and the behavior of SCOBY in addition to the mechanism of BC formation. Generally, the thickness of BCM, the difference among SWM (1, 4 and 5) was clear and differ from the same in WM after 15 days of incubation, where it was generally thick in SWM more than WM. The thickness was increased by the end of incubation (21 days) for SWM, while stop for WM SWM, while stop for WM (Fig. 3- D1 & 2).

A special shot was captured for BCM on HS medium after 21 days, where the membrane was raised upward against gravitation forming an air vacuole between the surface of HS medium and BCM which make oxygen available. At the same time, the SCOBY was extended in aggregates like spindle threads from the surface to the bottom of the vessel (Fig. 3- D3 a & b).

During three months of incubation the SCOBY growing in SWM still have the ability to form new BCM (Fig. 3-D5 a & b). It was mostly obvious in SWM containing FOW/E (SWM5) as it appears as distinct BCM layers (red arrow) followed by SWM 4 then a lower formation in the control medium without FOW (SWM1). While, no more BCM were detected at all in WM (4 & 5) and just a very thin transparent film of microbial growth was formed on the surface of the medium (WM1).

## Discussion

During the frying process there is a set of various chemical reactions occurs such as; oxidation, hydrolysis, isomerization and polymerization under the influence of high temperature, oxygen and moisture. The breakdown of oil constituents may lead to the formation of acids, alcohols, ketones, aldehydes, acrylamide, triglycerides, free and *trans* fatty acids, glycerol esters and its oxidized or polymerized forms [46, 47]. These compounds may be utilized as carbon source for BC production or initiate its synthesis. The current study aims to use frying oil wastes (FOW) as an inexpensive, green and sustainable source for BCM production. Several species of microorganisms are able to use triglycerides present in untreated cooking oil wastes as carbon source for growth and production of added-value compounds [48].

The data (Table 2) revealed that addition of 1% FOW onto the BC production medium resulted in a significant increase of BCM dry weight by 12.1% and 28.6% with increasing in BC yield from 4.7 to 4.6% and from 0 to 2.9%. in SWM and WM, respectively. While further increase in FOW concentration (2–5%) showed a significant decrease in BCM production. This finding is in agreement with a previous study which demonstrated that the addition of 1% rapeseed oil to culture medium enhance the cellulose biogenesis using *Komagataeibacter xylinus* by 604% and 650% of the wet and dry weight, respectively, while the addition of 2.5% or more of oil leads to decrease in the BC yield [40]. Generally, the first step in the biodegradation of vegetable oil is the cleavage of carboxyl ester bonds in tri-, di-, and mono-acylglycerols; which represents the major constituents of animal, plant, and microbial fats and oils; into fatty acids by lipases and esterases enzymes that produced by a wide range of microorganisms. This step is followed by degradation of both saturated and unsaturated fatty acids via a* β*-oxidation process [49, 50]. A previous study demonstrated that *Acinetobacter junii* WCO- 9 was efficient in degrading a variety of edible oils by p-nitrophenyl decanoate (p-NPD) enzyme and using it as a sole carbon source [31]. This may explain the current formation of thin BCM membrane on the surface of sugar free media containing FOW as a sole carbon source, where the SCOBY used for BCM production was consisting of a mix of *Acinetobacter lowffii* and *Candida krusei*. However, the presence of high concentrations of FOW may provide anaerobic conditions that prohibit the multiplication and growth of SCOBY and subsequentlly, affect the BCM production.

To confirm the ability of SCOBY to use FOW as a C/N source for BC production, a standard cellulose production medium (HS medium) was used where, carbon and nitrogen sources (glucose, yeast extract and peptone, respectively) are substituted by 1% FOW. The aim of this experiment is to find out whether FOW is used by SCOBY as a source of carbon/nitrogen to produce BCM, or does it just penetrate into the layers of cellulose during its formation. The obtained data indicated the difference between the addition of 1% FOW on HS and SW media (Table 3 and 4, respectively).

Moreover, it was noticed that the carbon source (represented in glucose) is more critical in the production of BCM than nitrogen source (peptone and yeast extract) as shown in the dry weight of BCM in HS (I) and (IV) compared with HS (II) and (III) media and at the same time explain the high BCM dry weight in sugared medium (SWM) compared with sugar free medium (WM). Also, it was indicated that FOW in this form is so difficult to be used as a sole carbon source for sufficient BC yield so, it was logically to transfer FOW into a simplified form by emulsification in order to increase its consumption as a carbon source. At the same time, it is important to increase the incubation period and study its effect on BC production.

Simply, emulsification is the process by which water is dispersed into oil in the form of small droplets in which the properties of this emulsified oil are very different from the starting oil [51]. The main function of adding emulsifier (emulsifying solvent) is to enhance contact between oil and water which can be measured by the emulsification index (EI). If the value of emulsification index is high, the contact between oil and water is also high and vice versa. In the current study, the data proved that using Tween 80 (5%) and ethanol (10%) in the ratio of 2:1 (FOW: Emulsifier) resulted in the optimum emulsification where the EI24 was 70% (Table 3). This result is in agreement with a study concluded that the use of tween 80 and ethanol can form a micro-emulsion system of eucalyptus oil, and the increasing concentrations of the combination of tween 80 and ethanol can increase the stability of the physical properties of the micro-emulsion system [52]. Meanwhile, the addition of surfactants or emulsifiers, such as Tween 80, enhance the bioavailability and solubility of cooking oil wastes [53–56].

On the other hand, ethanol as the other component of emulsifying solvent plays a critical role in both BC biosynthesis and FOW solubility. It was reported that supplementation of the growth/production media with alternative substrates like ethanol, improve BC biosynthesis [57–60]. Moreover, Molina-Ramirez et al. [58] concluded that the addition of alternative energy sources, such as ethanol to HS medium resulted in the growth of bacterial cellulose yield up to 279%. Also, the probability effects of ethanol on BC production was collected and discussed in our previous study [41].

The depletion of carbon source in the medium during the incubation period is considered the main factor in stopping the BCM production, where it was consumed in the growth of SCOBY cells and in the biosynthesis of BCM as well. From other view, Ross et al. [61] indicated that 40% of glucose may metabolized for gluconic acid byproduct while only 19% of glucose was incorporated into BC synthesis. In this regard, the presence of 1% ethanol (95%) may prevent the formation of gluconic acid [62] and subsequently enhance the BC biosynthesis.

In the current study, the presence of emulsified oil (FOW/E) with increasing the time of incubation into 21 days may help in enhancement the BCM production by 40.4% compared with 15 days (Table 5). The previous studies proved that glucose was consumed early in the first 11 days of incubation which represents the period of BC biosynthesis, while 50% of glucose was metabolized during the first 3 days in the initial BC layer formation [40, 63, 64]. This finding is not in agreement with our current results as the SCOBY starts the BCM formation in the eighth day (in SWM supplemented with FOW/E) compared with the control SWM which start the initial layer of BCM on day five and extend to 21 days in both media with increasing in BCM thickness. We can conclude that, the presence of emulsified FOW in addition to sugar and the presence of ethanol in the emulsifying solvent may help in maintaining the carbon sources, improving the BC biosynthesis and extend the incubation period.

On the other hand, the addition of low concentrations (1–2%) of FOW/E enhanced the BCM production by 32.6% compared with control but the high concentration resulted in gradual decreases reaching (Table 6). The accumulation of high concentrations of FOW/E on the surface of the medium causing a reduction in the oxygen concentration which required for growth and BCM production. In this regard, SCOBY tends to consume the carbon source (sugar and FOW/E) in growth and reproduction more than in the production of BCM to overcome this unfavorable anaerobic conditions which reflected in decrease in BCM dry weight. The statistical analysis confirmed this interpretation where the percentage of decrease in BCM dry weight with increasing FOW/E concentration from 1- 5% reached (39%). This is not agreed with a study revealed that although the availability of oxygen is one of the critical factors required for BCM production but *Komagataeibacter xylinus* was able to synthesize BCM for a longer time and at a greater distance away from the normal oxygen-rich surface interface in the oil- supplemented medium [40]. This point will be covered later on according to the current observations.

At the end of incubation period and harvesting of BCM, it was noticed that at higher concentrations of FOW (> 2%) some oil residues which did not consumed were still present in the medium (specially WM). While, there was a small amount of oil residues were present when using 1% FOW which explained in this case by the consumption of most amount of oil either in BC biosynthesis or incorporated into BC matrix during formation. It is worth mentioning that, some of the oil may be lost by stuck on the surface of the BCM so, cannot be estimated but it will be disposed during purification process. It is clear from the data (Table 7) that there is a variation in consumption between the different samples, where the emulsification of FOW and the presence of sugar in the medium help in the utilization of FOW and formation of BCM more than the original FOW and sugar free medium (86.5% and 39.2%, respectively). A previous study mentioned that the addition of emulsions may dramatically increase the area of the oil–water interface, thereby enhancing its bioavailability [65].

After harvesting of BCM at the end of incubation period, it was noticed that there is a difference between the addition of FOW only and FOW/E, where the low concentrations of FOW was incorporated onto the BCM matrix while the high concentrations causes a change in the surface of the BCM (making small holes and cavities). Whereas, by the addition of FOW/E it was penetrated into the BCM and appear as homogenized in its fabrication matrix (Fig. 2b).

A preliminary experiment was conducted in the beginning of the study before determination of the optimum conditions of FOW concentration, incubation period, emulsifying ratio and concentration. Aiming to investigate the effect of gamma irradiation; as a pretreatment step; on the FOW and its availability for utilization as a sole source of nutrients for growth and BC biosynthesis by SCOBY. The preliminary results of irradiated FOW (0, 10, 20, 40, 50 kGy) showed the formation of oil aggregates; resemble elastic film; on the surface of the sugar free (W) medium after 15 days of incubation (Fig. 2a).

This result can be explained as; *i):* the amount of FOW used (5 ml/300 ml W medium which represents 3.3%) was high enough to create an anaerobic conditions by accumulating as a layer on the surface of the medium that prohibit the BC biosynthesis- *ii):* the FOW need to be emulsified at first to increase its availability- *iii):* the FOW cannot be used as a sole carbon source and the medium needs to provide with other source of carbon for the growth and production of BCM- d): the irradiation doses may affect the structure of FOW and resulted in the formation of undesirable substances that not suitable as nutrients for the growth and BCM production. Whatever, further studies are needed because the literature has insufficient data about the effect of gamma radiation on the edible plant oil itself, where all the previous researches reported the effect of radiation on the oil content of the seeds before and after irradiation.

In this regard, it was planned to conduct the experiments as mentioned above in the manuscript in addition to demonstrate the effect of radiation of emulsified FOW on BCM production. As a general, the data proved that a significant difference between irradiated and non-irradiated FOW/E was occurred according to the difference in BC dry weight (Table 8). The increase (2.5%) was recorded at dose 10 kGy compared with non-irradiated FOW/E, while recorded 34.1% increase compared with SWM without FOW/E. Nothing was present in the literature to explain this result except related articles that revealed that; gamma irradiation at 10 kGy, has a positively impact in biomolecules like lipids, carbohydrates, proteins, and other phytochemicals by inducing structural and chemical changes, where it accelerates lipid oxidation leading to increased values for fatty acid. Whereas, increasing γ-irradiation induces changes in fatty acid and amino acid composition as illustrated in soybean oil and peanut [66, 67]. However, soybean oil extracted from soybeans subjected to gamma radiation up to 10 kGy exhibited no significant changes in various physicochemical characteristics, including lipid content, fatty acid composition, acid value, peroxide value, and trans fatty acid content [68]. On the other hand, it was found that gamma irradiation (3 kGy) caused a significant decrease in fat content and a significant increase in saturated fatty acids/unsaturated fatty acids ratio of cocoa bean samples, while fatty acid composition changed with increasing the irradiation dose [69]. However, the irradiation doses may affect the FOW/E which recommend further studies to follow the reactions and the changes in its composition.

As this is the first investigation study the capability of using FOW as a carbon source for BC production, so the process was followed and documented with photo-shoots along the incubation period. As a general, BCM is formed on the surface between medium and air interface where the oxygen is available [70]. Based on the principle of the downward force of gravity, it is logically that BCM will be sink into the bottom of the vessel once formed as it possesses a higher weight and density compared to the medium. However, a previous study reported that there is a frictional force that arises between the cellulose membrane and the wall of the glass vessel against gravity that prevents the membrane from falling downward and allows it to float on the surface of the medium. The thickness of BC membrane increase along the incubation period and creating anaerobic conditions that prevent the formation of more layers of cellulose [71]. On the other hand, it was documented that the presence of oil in the medium prevents or reduces the formation of this force between the cellulose membrane and the vessel wall, causing the layers of BC formed to fall towards the bottom of the vessel due to gravity. This provides the chance to form another layers of cellulose due to the availability of aerobic conditions [40]. Whereas, the current study showed different mechanisms through capturing some documented moments while tracking the formation of the cellulose membrane in the presence of FOW in both its emulsified and non-emulsified forms. It was found that the process of forming the cellulose membrane depends on the distribution of FOW on the surface of the medium, hence the symbiotic culture adapts and behaves accordingly. In its non-emulsified form, it forms oil spots of various sizes that spread randomly across the surface of the medium or adhere to the walls around the perimeter of the vessel. While the presence of oil in emulsified form makes it more homogenous and spread as a uniformly and consistently layer on the whole surface. In addition, the method of forming the cellulose membrane varies according whether it is a sugared water or sugar free medium as will have discussed below.

It is well known that microorganisms grow in the broth medium after inoculation according to their oxygen needs. In the aerobic species, growth should be near from the surface where atmospheric oxygen is available, while anaerobic species grow at the bottom. Whereas, in the current study the growth of the symbiotic culture was illustrated in different ways as described in table (9). The results demonstrated that there is a change in the behavior of SCOBY related to the constituents of the media. In Sugar free medium (WM1), the growth was rapid and tenuous which referring to the lack of nutrients required for their growth. In the first days may be they have forced to use the traces of nutrient that were transferred during the inoculation process from the pre-inoculum medium. After exhaustion of nutrient residues (on the third day), the symbiotic culture forms a strand of cells extending in a concave shape from one side of the vessel wall to the opposite side (Fig. 3B1 a). This observation was interpreted as an attempt of the symbiotic culture to survive longer, where the aggregation of cells in the form of clusters have a stronger bonding than individual cells and thus a greater ability to survive under abnormal conditions. This phenomenon was recorded in the same manner when adding non-emulsified FOW (2%) to the sugar-free medium, where the same concave bridge appeared between the edges of the container from two opposite sides (Fig. 3 B1 b). Nothing was found in the literature to explain this phenomenon in clear manner but it was reported that some *Acinetobacter* sp. possess an auto-agglutinating nature and noteworthy adhesiveness to various hydrophobic and hydrophilic abiotic surfaces like plastics, glass and stainless steel in which aggregating bacteria are protected from environmental stresses and also forming the first steps in biofilm formation [72, 73]. Generally, it was observed that the growth of the symbiotic culture occurs more rapidly in a sugar-free medium, then eventually stops increasing and remains in the form of a thin film floating on the surface of the medium which falls to the bottom of the vessel when stirred manually at the end of the incubation period.

From a physiological view and according to the previous knowledge, bacteria can behave two ways under abnormal conditions; like unfavorable temperature or pH and scarce or absence of nutrients; by entering in a dormant state or form spores. It was found that non-sporulating *Bacillus subtilis* cells follow an alternative strategy nominated as"oligotrophic growth rate", where they can be alive in pure water for many months tolerating different stresses by adapting their cells into coccoid and slow down their growth rate in order to survive under deep starvation conditions [74]. Another study explained that the metablome and trancriptome data of *E. coli* cells that transferred from a high level glucose medium to a glucose free medium proved that, the non-growing bacteria can retain a high metabolic activity by feeding on internal resources like; proteins, glycogen and RNA [75]. For instance, glycogen utilization was responsible for providing the cells with energy during the early stage of carbon starvation. Moreover, the metabolism and death rate during carbon starvation depends mainly on their rate during pre-starvation, where the slower growth leading to exponentially slower death rate [76]. They concluded that *E. coli* survive longer if it previously grew slower which explain the trade-off between fast growth and longer survival which means a compromise between growth rate and death rate, that put bacteria under strong pressure obligate it to grow slow. In this context, a study confirms that *E. coli* can maintain its viability in starvation by feeding on amino acids derived from dead cells [77–79]. At the end of the incubation period and shaking the vessel, the BCM sink into the bottom of the vessel in the control medium (WM1 & SWM1), while still floating on the surface of both sugared and sugar- free media and in the presence of both emulsified and non-emulsified FOW. This may be referred to the incorporation of some oil droplets into the BC matrix during its formation helping to reduce its weight and thus preventing it from settle to the bottom of the container (Fig. 3 E1&2). It was shown that when using the standard BC production medium (HS medium) under the same growth conditions and without the addition of FOW, there was a difference from the theory mentioned in a previous study [71] and contrary to the findings illustrated before [40]. It was found that the BCM formed was raised upwards from one of the sides adjacent to the vessel wall against the force of gravity, creating an air gap like a vacuole (white arrows in Fig. 3 E1-a). In addition, the growth of the symbiotic culture in the form of dense intertwined threads was observed which acting as a lifting force that may unexpectedly pushes the BCM upwards (purple arrow in Fig. 3 E1-b). In contrast, the control SWM (without FOW) showed an opposite phenomenon to that mentioned above in HS medium where the BCM was submerged from one side into the medium (yellow arrow in Fig. 3 E2-b) while the facing side still adhere to the vessel wall (orange arrow in Fig. 3 E2-b). This action resulted in the provision of new surface area in addition to oxygen availability that allowed the formation of new layers of BCM. According to a previous study, there is a relationship between the microbial activity and the growth rate which affected by the availability of nutrients under suitable and unsuitable environmental conditions [80]. The previous studies on microorganisms have been mostly investigated on the lab under optimum or near-optimum growth conditions, while poor information in the literature is available about the behavior of microorganisms at slow-growing states (i.e., near-zero growth and maintenance metabolism). Based on the above, the current study may pave the way towards more investigations in order to understand the mechanism, physiological response and behavior of microorganisms under limitation of growth conditions which still unexplored field in microbiology.

## Conclusion

The present work represents a preliminary study that proved the capability of SCOBY for utilization of FOW in its emulsifying form as a carbon source for BC synthesis which achieved the hypothesis of the study. By comparing the current results with the previous studies that using other alternative carbon sources for BC production, it can demonstrate that: (i): in terms of production (gL- 1); the range of BC production (5.4 gL- 1) is compatible with the range that obtained in the previous studies [17, 81] using different agro-industrial wastes (0.3–20 gL- 1), (ii): in terms of cost; both current study and the previous studies are supporting the circular economy principles by converting wastes into valuable products, (iii): in terms of availability and sustainability: FOW is an abundant source that is available all the time due to its association with the increasing consumption by the growing population which means the continuity and sustainability of BC production. Accordingly, bioremediation is one of the potential scenarios for the application of the current findings in real-world by removing frying oils polluted domestic and industrial wastewater. On the other hand, it can be applied in the lab scale to understand the behavior of the BC producing microorganisms; especially SCOBY; in the environment under stressed conditions. Ultimately, using of FOW during BC production may affected its properties which needs more characterization and specific study in details in order to recommend its potential applications.

### Limitation of work

The limitation of work in the current study and how to overcome in the future research were concluded as follows:

1. Using of one type of FOW from one source was not a representative sample, while testing different FOW from different types and sources is more preferable.
2. The number of replicates used in the designed experiment was duplicates, whereas using triplicates or more would reduce the errors and ensure the validity and reliability of the results.
3. There was a lack of information on the effect of gamma irradiation on the edible oils including frying oils and their wastes since most previous studies were reported the effect of gamma irradiation on the plant seeds and their oil content. Further investigations on the effect of different doses of gamma irradiation on the microbiological and physiochemical properties of the different type of oils were recommended.
4. The mechanism of degradation or utilization of FOW using a symbiotic culture was unavailable in the literature which may differ from the way of single strain. These requires more studies to understand its behavior and recognize the exact pathway.
5. The specific manner that the BC producing strains were follow in the formation of BC membrane on different media was not investigated before, which needs more studies and documentation.

### Future work

There are many ideas will be implemented in consistent manner based on the results and information that have been obtained in order to develop new techniques of BC production, modification and applications.

## Supplementary Information

Additional file 1. The comparison among bacterial cellulose formation within different media containing emulsified and non-emulsified FOW was attached as an additional pdf file (additional file 1) titled as: Table 9: The process of BCM formation on the surface of the SWM and WM in the presence of FOW and FOW/E.

## Data Availability

The data (Table 9) that supporting the results and conclusion of the current study is attached as an additional file. All other data is included within the manuscript. Any additional information or quires will be provided by sending an e-mail to the corresponding author.

## Abbreviations

BC: Bacterial cellulose
BCM: Bacterial cellulose membrane
SCOBY: Symbiotic Culture of Bacteria and Yeast
SWM: Sugared water medium
WM: Water medium (Sugar free)
kGy: Kilo gray
FOW: Frying oil wastes
FOW/E: Emulsified frying oil wastes
WCO: Waste cooking oil
FFA: Free fatty acid
PHA: Polyhydroxyalkanoate
EI: Emulsification index
