# Supplementary material for: Utilization of gamma irradiated emulsified frying oil wastes as a carbon source for sustainable and economical production of bacterial cellulose membrane
Source: BMC Microbiol. 2025 Apr 24;25:242. doi: 10.1186/s12866-025-03931-7 (PMC12020031; doi:10.1186/s12866-025-03931-7)
Supplement: Supplementary file 1 — Additional file 1. The comparison among bacterial cellulose formation within different media containing emulsified and non-emulsified FOW was attached as an additional pdf file (additional file 1) titled as: Table 9: The process of BCM formation on the surface of the SWM and WM in the presence of FOW and FOW/E. [file 12866_2025_3931_MOESM1_ESM.pdf]

Table (9): The process of BCM formation on the surface of the SWM and WM in the presence of FOW and FOW/E

Comparing Items	Sugar-free medium (WM1)	Sugared-water medium (SWM1)	Sugar-free medium (WM4)	Sugared-water medium (SWM4)	Sugar free medium (WM5)	Sugared-water medium (SWM5)
Presence of FOW	No FOW	No FOW	1% non-emulsified FOW	1% non-emulsified FOW	1% FOW/E	1% FOW/E
Appearance of the media	Clear and slightly turbid after inoculation by SCOBY.		FOW was adhered to the wall of the vessel, with some oil spots in varying sizes which were distributed on the surface of the medium.	FOW is mainly distributed at the edges along the wall of the glass vessel and as a large irregular spot in the center with some spaces devoid of oil.	In the presence of emulsified FOW, the oil layer appeared more homogeneous in both the sugar-free and sugared water medium (WM5 & SWM5).	
Behavior of SCOBY	- SCOBY grew on the bottom of the vessel and as tiny aggregates then, form a very thin layer on the surface during the first and second days.<br>- SCOBY forms a strand of cells extending in a concave shape from one side of the vessel wall to the opposite side in the third day.<br>- The growth of the symbiotic culture occurs more rapidly in a sugar-free medium, then eventually stops increasing and remains in the form of a thin film floating on the surface of the medium.	- The SCOBY began to grow on the bottom of the vessel after 24 hrs then appeared on the surface of the medium as a very thin membrane starting from the third day.<br><br>- It was noticed also, the presence of microbial growth on the bottom of the vessel during the incubation period	SCOBY begins to grow in two different ways:<br>*a.* after 48 hours grew in a cord likes a ribbon that extended from the bottom of the vessel to the central empty space between the oil spots on the surface of the medium (Figure 3 B2 a). After 6-12 days, it appears as 3 branches joint together and extend to 3 cm under the surface of the medium (Figure 3 B2 b).<br>b. The SCOBY adhere to the vessel's wall in an oil-free area allowing its attachment, growing, and forming a BCM as shown in figure 3 (C1 a, b & c).	-The symbiotic culture begins to grow in the bottom of the vessel in addition to the whole medium that appeared turbid after three days of incubation.<br><br>-On day eight, microbial growth aggregates were observed in the sites free of oil spots on the surface of the medium.	The SCOBY grew on the bottom of the vessel in the whole surface within the first three days of incubation.	
Process of BCM formation	- NO BCM formed	- BCM began to be formed almost in a consistent and uniform manner across the entire surface area of the medium in the 5th day before the other treatments.	- In the first type:<br>BCM starting formation in central space among oil droplets.<br>- In the second type:<br>BCM starting formation in the point of attachment of SCOBY to the vessel wall, then distributed as a thin layer on the surface of the medium.	BCM formed randomly in the free spaces among the FOW spots as shown in figure (Figure 3 D2).	- The BCM was formed in 8th day as a uniformly layer distributed on the surface of both media with some oily bubbles appears within its matrix.<br>- BCM appeared thicker in SWM5 compared with all other BCM even in the control SWM.	
At the end of Incubation	The thin growth layer of SCOBY fell to the bottom of the vessel when stirred manually at the end of the incubation period..	BCM was sinking into the bottom of the vessel,	At the end of incubation period and shaking the vessel, the BCM was still floating on the surface of both media. (Figure 3 E1&2).		BCM was still floating on the surface of both media supplemented with emulsified FOW. (Figure 3 E1&2).	

SWM1 & WM1: Sugared water medium (6% sugar) and Sugar free medium (water only) + SCOBY as a control medium.
SWM4 & WM4: Sugared water medium (6% sugar) and Sugar free medium supplemented with 1% FOW (non-emulsified) + SCOBY.
SWM5 & WM5: Sugared water medium (6% sugar) and Sugar free medium supplemented with 1% FOW (non-emulsified) + SCOBY.
